# Adherence to evidence-based practice across healthcare professionals in Germany: results from a cross-sectional, nationwide survey

**DOI:** 10.1186/s12913-022-08682-z

**Published:** 2022-10-25

**Authors:** Katja Ehrenbrusthoff, Tobias Braun, Carolin Bahns, Lisa Happe, Christian Kopkow

**Affiliations:** 1 Department of Applied Health Sciences, Division of Physiotherapy, Hochschule Für Gesundheit (University of Applied Sciences), Gesundheitscampus 6-8, 44801 Bochum, Germany; 2 HSD Hochschule Döpfer GmbH (University of Applied Sciences), Waidmarkt 3 & 9, 50676 Cologne, Germany; 3 Department of Therapy Science I, Brandenburg University of Technology Cottbus – Senftenberg, Universitätsplatz 1, 01968 Senftenberg, Germany; 4 Department for Health Services Research, Assistance Systems and Medical Device Technology, Carl Von Ossietzky University Oldenburg, Ammerländer Heerstr. 114, Oldenburg, Germany

**Keywords:** Evidence-based practice, Evidence-based medicine, Healthcare professional, Exploratory regression analysis, Physical Therapy, Occupational therapy, Allied health professions

## Abstract

**Background:**

Adherence to evidence-based practice (EBP) is considered a key competence to improve healthcare quality. In this study, we aimed to describe the EBP adherence of healthcare professionals working in Germany and to explore barriers and facilitators regarding the implementation of EBP in clinical practice.

**Methods:**

A nationwide online survey was performed among healthcare professionals in Germany from January to April 2018 using the German version of the Evidence-based Practice Inventory (EBPI) questionnaire for a comprehensive assessment of adherence to EBP. Univariate logistic regression analyses were performed to explore the association between demographic and professional determinants and each EBPI dimension.

**Results:**

We analysed data of 889 participants, including 318 physical therapists, 154 occupational therapists, 137 midwifes and 280 participants of six other healthcare professions. Approximately 70% of the participants generally demonstrated a positive attitude towards EBP and believed that EBP was useful in clinical practice. Broadly, 80% of the respondents evaluated themselves as being able to enact EBP behaviour in clinical practice. In contrast, less than 70% preferred to use quantitative information instead of their intuition to inform their habitual clinical behaviour. Still, 20 to 30% reported that EBP did not sufficiently account for their clinical experience and differences between patients.

The strongest facilitators to EBP adherence across at least three dimensions of the EBPI were the availability of ≥ 60 min for scientific literature at work (OR: 9.67; 95% CI: 5.86; 16.30), followed by a master or higher academic degree (OR: 9.09, 95% CI: 5.86; 14.28) and the involvement in ≥ 1 scientific publication (OR: 7.06, 95% CI: 5.10; 9.85).

**Conclusions:**

This study showed that healthcare professionals in Germany in general had a positive attitude towards EBP although they currently do not consider EBP principles in its entirety. The most important determinant positively influencing a healthcare professional’s decision to perform EBP was the time available for scientific literature at work. German healthcare professionals experience similar barriers towards the implementation of EBP in clinical practice compared to other international healthcare settings. These barriers should be targeted by future research.

**Trial registration:**

German Clinical Trials Register (DRKS00013792). Registered 19 January 2018.

**Supplementary Information:**

The online version contains supplementary material available at 10.1186/s12913-022-08682-z.

## Background

The use of an evidence-based approach in clinical practice is accepted as a core competence across most health professions [1]. Decisions in clinical practice, based on research evidence as well as a clinician’s expertise and patient preferences and contextual resources, improve patient outcomes and help to reduce variations not explainable by need or preferences of certain populations [2] in clinical decisions across healthcare professions and settings [3]. Despite this accepted notion, the implementation of research evidence into clinical practice still varies greatly among healthcare professions, especially regarding the extent as to which research evidence currently informs their clinical decision making [4]. For the improvement of clinical care, the gap between clinical practice and research evidence needs to be narrowed, e.g. by using an evidence-based practice (EBP) approach [5].

EBP has been initially defined as a ‘problem solving approach aiming to improve the quality of patient care by informing clinical decision making in patient care by the current best evidence’ [5]. Still, the creation of clinical settings and requirements that facilitate EBP for healthcare professionals who wish to provide high-quality patient care remains a major challenge [6].

Across many settings and professions, a wide range of studies reported barriers and facilitators regarding the implementation of research evidence into clinical practice [7]. Major barriers and facilitators identified were related to the clinician’s individual evaluation of the EBP concept, perceived individual competencies to perform EBP, or an individual’s estimation in how far an EBP culture or conform behaviour was assumed by superiors or colleagues [8].

Acknowledging that EBP is influenced by several and individually varying factors, the evaluation and implementation of EBP remains challenging. The ‘Evidence-based Practice Inventory (EBPI)’ is a questionnaire developed to assess the construct of ‘EBP adherence’ and to identify barriers and facilitators for EBP across a variety of healthcare professions and settings [9]. It can be used to assess EBP adherence under local conditions to provide insights into potential barriers and facilitators, which could then be used to develop strategies for a further evaluation and implementation of EBP.

In Germany, training of most allied healthcare professions is or has been mostly performed at vocational schools and not at higher education institutions [9]. Although physicians and psychologists are trained at higher education institutions, other professions such as physical therapy, occupational or speech therapy remain mostly at vocational schools. Training of nursing is being performed at least partly at higher education institutions, and for midwifery a recent EU legislation caused change from vocational schools to education of midwives at tertiary institutions [10]. Due to different academic and non-academic training routes, imparting of EBP knowledge as part of the formal training is different [11]. However, incorporating the best research evidence into clinical practice to ensure high quality patient care needs to be ensured.

## Methods

This study aimed to describe the EBP adherence of healthcare professionals working in Germany and to identify barriers and facilitators regarding the implementation of and adherence to EBP in clinical practice.

The guidelines for Reporting of Observational Studies in Epidemiology (STROBE) [12] and the Checklist for Reporting Results of Internet E-Surveys (CHERRIES) [13, 14] were followed in the reporting of this study. The reporting of statistical results was further informed by the ‘Statistical Analyses and Methods in the Published Literature’ (SAMPL) guidelines [15].

### Study Design

The present report is based on data from a cross-sectional study which aimed to (a) evaluate the measurement properties of a German language version of the EBPI [14] and (b) to identify barriers and facilitators in the implementation and adherence to EBP. Details of this study have been reported elsewhere [14, 16]. Briefly, the EBPI was completed twice in an online survey by healthcare professionals in Germany. In the present study, we used only the baseline data (first assessment of the EBPI).

### Participants

This online survey was made accessible to all German-speaking healthcare professionals via an unconfined public internet link and by means of convenience sampling. Only data from adult (≥ 18 years) healthcare professionals (e.g. medicine, midwifery, nursing, occupational therapy, speech and language therapy, sports therapy, psychology, physical therapy), predominantly working in Germany were included. Questionnaires from respondents (a) who stated to work < 1 h per week with patients or in clinical care, (b) who terminated the survey before completing the 12^th^ screen and, (c) those who did not fill in any of the five dimensions of the EBPI [14].

For the recruitment of healthcare professionals, miscellaneous media and communication paths were employed. We conceptualized an invitation letter, a statement to the press and a short “advertisement”, all comprising an outline of the study aim and procedure as well as contact information. We informed the health care professionals community in Germany about the survey by means of publication via different media by the institutions, professional societies and journals (See Additional file 2) and asked to spread the survey link and the project description to as many colleagues as possible (“snowball principle”). We offered no incentives for participation [14]. A total sample size of 800 participants was targeted per protocol for the analyses of measurement properties of the EBPI [14]. However, it was intended to include as many participants as possible and therefore, a maximum sample for the survey was not defined a priori.

### Data collection

#### Survey instrument

EBP adherence among healthcare professionals working in Germany was assessed using the EBPI, an instrument covering five dimensions with 26 items in total [4].

1. ‘Attitude’ (a clinician’s individual evaluation of EBP)
2. ‘Subjective norm’ (a clinician’s own estimate of the social pressure to perform or not to perform EBP behaviour)
3. ‘Perceived behavioural control’ (the extent to which a clinician feels able to enact EBP behaviour)
4. ‘Decision making’ (the extent to which new information reshapes the clinician’s current understanding and (habitual) behaviour)
5. ‘Intention and behaviour’ (the clinician’s aim and actual response, respectively, to apply EBP) [4, 17–19].

Respondents rated 26 statements (items) on a scale ranging from 1 to 6 with two antonyms (e.g. *useful* versus *useless* or *ability* versus *inability*). Based on the operationalisation by Kaper et al. [4], the term ‘EBP adherence’ was defined as the overall self-reported estimation a health professional provided across all dimensions, which together capture EBP as a complex construct [1, 14].

#### Online Survey

We performed an online survey among healthcare professionals in Germany, set up by using the software “SoSci Survey” (SoSci Survey GmbH, Munich, Germany; https: //www. soscisurvey.de). The survey could be accessed online only via an internet link to a “survey homepage” [14]. The survey comprised 14 pages. Participants had the opportunity to provide socio-demographic data if they so wished [14]. This freedom of choice was decided to ensure anonymous participation and to support survey completion. These characteristics were defined a priori as potentially influencing factors for the adherence to EBP based on findings from the current literature [20–23]. These were: age, sex, profession, highest professional degree, contact time with patients per week, primary setting of work, work experience, size of community/municipality of employment, federal state of employment, hosting of lectures or workshops on EBP, the availability of scientific literature at work, involvement of ≥ 1 scientific publication, available time for studying scientific literature at work per week [4]. The full German language EBPI was presented with one screen displaying each scale dimension. The survey’s items were provided in a standardised, unchanged sequence and participants had the possibility to review and change their answers at any time before survey termination (See Additional file 1 for an English version of the online survey) [14].

The survey was conducted from 25^th^ January to 15^th^ April 2018.

### Data analysis

Descriptive statistics (such as mean values with standard deviation and range) were employed to present sample characteristics. Missing values were not imputed. Neither a weighting of items nor propensity scores were used [14].

The 6-point Likert scale response options of the EBPI were dichotomized for further analyses. A rating of point 1 to 3 was rated as an unfavourable answer (e.g. ‘not capable of’), whilst a reporting of points 4 to 6 was viewed as a favourable answer (e.g. ‘capable of’). For example, for dimension 1, item 1 was coded as „useless “ and „useful “, whereas item 5 was coded as “disregards” and “respects” as antonyms.

A favourable evaluation of EBP was further operationalised by considering the sum scores for each dimension separately. The cut-off value for dichotomisation was chosen as the lowest value of the third quartile of the sum score per dimension [24, 25]. For instance, dimension 3 (perceived behavioural control) comprised items 14 to 19 (6 Items), hence the maximally attainable sum score was 36. A clinician was defined as estimating himself being able to enact EBP (favourable evaluation of EBP) when the sum score of items 14 to 19 equalled or was greater than 75% of the study population’s sum score for this dimension. With this approach, a population specific description of results was intended, facilitating future comparisons across different populations [25].

Univariate logistic regression analyses were performed to explore the association between each potentially influencing factor and each EBPI dimension. An univariate logistic regression model was chosen rather than a multivariate approach to present results at its most modest level so as to provide a basis for future hypothesis generation [26]. Influencing factors were identified and incorporated, when they have been used in prior studies assessing EBP adherence.

Odds ratios (OR) and their associated 95% confidence intervals (CI) were reported for each sub-category of the demographic characteristics. OR were defined to describe the likelihood of demonstrating a favourable evaluation of a particular dimension of the EBPI (e.g. dimension 2 ‘subjective norm’: the estimate of the social pressure to perform or not to perform EBP behaviour) given a particular characteristic (e.g. having more than 10 h contact time with patients). One sub-category of each characteristic was used as the reference against which the odds of all other sub-categories was calculated. The reference was either the greatest subsample or, for dichotomous determinants, the more likely characteristic variable in clinical practice (based on the evaluation of the author team, e.g., reference “no” for availability of scientific literature at work place) to arrive at the most informative interpretation of results.

For some of the demographic characteristics, where subsamples were considered to be too small, some categories were collapsed in order to allow further analysis using them as determinants in the logistic regression analysis. For example, only 40 individuals indicated to have a higher academic degree (PhD) as their highest professional degree which was decided to be combined with the “masters” category (*n* = 106). In addition, some of the demographic characteristics were not included in the regression analysis, as they were determined not to provide reliable results regarding a clinician’s evaluation of EBP (e.g. age, as inherently expressed in work experience).

Data from the online survey were stored and processed as SPSS and R data files by SoSci Survey. For data analysis SPSS Version 25.0 statistical software (SPSS Inc., Chicago, IL, USA) and R Version 3.5.0 (The R Project for Statistical Computing, Vienna, Austria) were used.

### Ethical considerations

Ethical approval was obtained from the ethics committee of the German association of physical therapists (Deutscher Verband für Physiotherapie e. V., Ethics Committee No.: 2017–13). Before taking part, participants provided written informed consent for data analysis and publication. The study was registered a priori in the German Clinical Trials Register (DRKS00013792).

## Results

### Participants

**Fig. 1:**
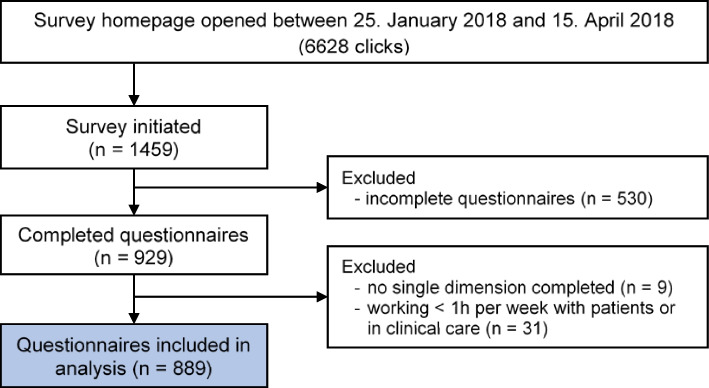
Flow chart of the study

The online survey homepage was accessed 6628 times and 1459 participants initiated the survey. In total, 889 questionnaires were finally included in the analysis (see Fig. 1 for details), resulting in an overall response rate of 13.4% according to the number of accesses to the survey homepage [14].

Six hundred fifty-seven (73.2%) of the 889 healthcare professionals involved were female. Participants were on average 37.4 ± 11.5 years old. In total, individuals from nine different healthcare professions took part in the survey with the group of physical therapists being the largest (n = 318; 35.8%). Most of the participants completed all dimensions of the questionnaire. Socio-demographic characteristics of the participants are provided in Table 1.

**Table 1:** Socio-demographic characteristics of the study participants (*n* = 889)

Characteristics	
**Age in years, mean** ± SD (range)	37.4 ± 11.5 (18 – 69)
**Sex: male/female/other, n (%)**	225/657/3 (25.4/73.2/0.3; *n* = 885)
**Individual number of professions: one/two/three, n(%)**	838/49/2 (94.3/5.5/0.2; *n* = 889)
**Professions†, n (%)**	
Physical Therapy	318 (35.8)
Occupational therapy	154 (17.3)
Midwifery	137 (15.4)
Speech and language therapy	79 (8.9)
Nursing	73 (8.2)
Medicine	28 (3.1)
Sport therapy/sport sciences	26 (2.9)
Psychology	9 (1.0)
Other	14 (1.6)
≥ 1 profession	51 (5.7)
**Highest professional degree, n (%)**	
Undergraduate	90 (10.1)
Diploma (vocational school)	368 (41.4)
Bachelor/diploma (university)	273 (30.7)
Master	106 (11.9)
Higher academic degree	40 (4.5)
Missing	12 (1.3)
**Contact time with patients, hours per week, mean ± SD (range)**	26.7 ± 11.8 (1 – 60) (*n* = 861)
**Primary setting of work, n (%)**	
Hospital	196 (22.0)
University clinic	58 (6.5)
Rehabilitation clinic	61 (6.9)
Outpatient clinic/private practice	468 (52.6)
Other	96 (10.8)
Missing	10 (1.1)
**Work experience in years, mean ± SD (range)**	13.3 ± 10.8 (0 – 44) (*n* = 870)
**Employment situation, n (%)**	
Employee/worker	584 (65.7)
Self-employed	159 (17.9)
Freelancer	83 (9.3)
Undergraduate/in training/practical year	59 (6.6)
Missing	4 (0.4)
**Leading/leadership position: yes/no, n (%)**	286/589 (67.3/32.7) (*n* = 875)
**Having inter-professional communication, n (%)**	769 (86.5) (*n* = 884)
**Inter-professional communication with other professions**^a^**, n (%)**	
Medicine	679 (88.3)
Nursing	461 (59.9)
Physical Therapy	454 (59.0)
Occupational therapy	345 (44.9)
Psychology	297 (38.6)
Speech and language therapy	270 (35.1)
Sport therapy/sport sciences	146 (19.0)
Midwifery	92 (12.0)
Other	96 (10.8)
**Size of the city/municipality of employment, n (%)**	
< 5.000 (rural community)	64 (7.2)
5.000 – 20.000 (small town)	153 (17.2)
20.000 – 100.000 (mean sized city)	235 (26.4)
> 100.000 (large city)	427 (48.0)
Missing	10 (1.1)
**Available time for scientific literature studies at work within a typical week in minutes per week, mean ± SD (range)**	53.6 ± 100.8 (0 – 900) (n = 751)
**Availability of scientific literature at work place, n (%)**	578 (66.5)
**Drafting of or involvement in ≥ 1 scientific publication, n (%)**	277 (31.2)
**Hosting of lectures or workshops on evidence based practice, n (%)**	120 (13.6)

Values are the total numbers (percent) or indicated otherwise. Mean values are given with the standard deviation (range). Different sample sizes within each sample due to missing values

^a^Multiple answers possible

#### EBP adherence of healthcare professionals

### Response pattern

**Fig. 2:**
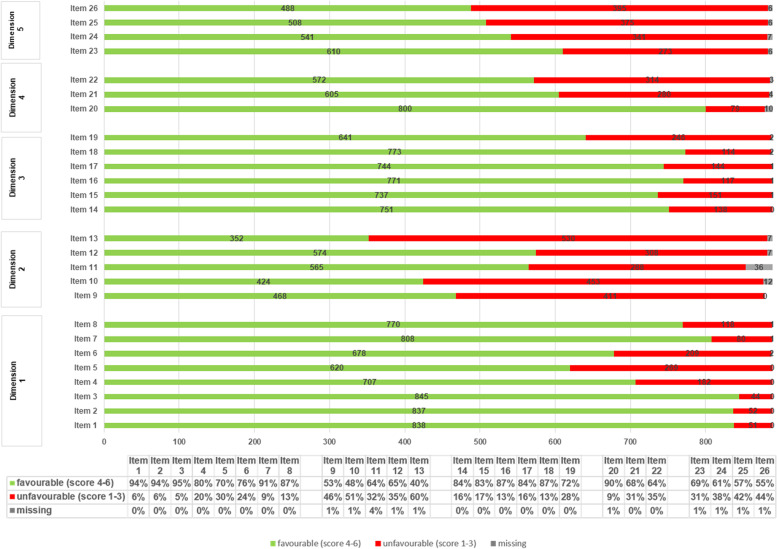
Response pattern per item

A comprehensive overview regarding the response pattern per item is illustrated in Fig. 2.

#### Dimension 1 – Attitude

The majority of respondents demonstrated a generally positive attitude towards EBP, as 94% accounted it as useful to improve their patients’ outcomes (item 1), 94% considered it an important aspect of high-quality care (item 2) and 95% as an improvement in their clinical decisions (item 3). 80% of the respondents stated that EBP respects their clinical experience (item 4). Respondents were more cautious regarding the evaluation as to whether EBP respects individual differences between their patients (item 5) or leaves them autonomous in their individual clinical decision (item 6). 91% rated EBP as helpful for better clinical decisions (item 7) and 87% judged clinical guidelines as helpful for making decisions (item 8).

#### Dimension 2 – subjective norm

Respondents were equally balanced (53%) as to whether colleagues discouraged or encourage them (item 9) or the department they are working in pays a lot of or no attention to the application of EBP principles in their clinical decisions (item 10) (48%). 64% of the respondents reported that managers in their department supported them to apply EBP principles in their clinical decisions (item 11), of which 78% (223 out of 287 respondents) simultaneously occupied a leadership position themselves. 65% stated that they frequently discussed and challenged how they make clinical decisions (item 12). In contrast, 60% stated that they rarely discussed research evidence from the literature with colleagues (item 13).

#### Dimension 3 – perceived behavioural control

Respondents felt similarly capable of applying the core principles of EBP in clinical practice and to critically appraise research evidence from scientific literature (item 14 and 17), as well as searching for evidence and translating it to the individual care of patients (item 16 and 18). 83% reported to be capable of translating information needs into relevant clinical questions (item 15). Of all respondents, 72% felt capable of keeping up with the latest research evidence from the literature (item 19).

#### Dimension 4 – decision making

90% gave a high priority to the thorough understanding of all relevant background information to answer individual clinical questions (item 20). 68% reported to be in favour of incorporating quantitative information (item 21) and 64% of using facts and arguments into their clinical decisions respectively (item 22).

#### Dimension 5 – intention and behaviour

Of all respondents, 69% stated to frequently use (item 23) and 61% to prefer to use research evidence for the support of individual clinical decisions (item 24). Response rates were nearly balanced as to whether asking colleagues or a literature search was preferred to find answers for clinical decisions (item 25) or whether available research evidence was incorporated rarely or frequently to answer clinical questions (item 26).

The response pattern regarding the overall favourable evaluation of EBP, dichotomised according to the population specific cut-off values (upper quartile – favourable evaluation of EBP) are detailed in Table 2. Across all dimensions, 26% to 30.0% demonstrated an overall favourable evaluation of EBP.

**Table 2:** Response pattern—overall favourable evaluation of EBP

EBPI Dimension	Nr of items	Scale range	Valid cases (Dimension fully completed)	Mean ± SD (population specific range)	Cut-off value favourable EBP evaluation (sum score)	Degree of favourable EBP evaluation, n (%)	
						Favourable	Non-favourable
1: Attitude	8	8–48	885	38.2 ± 6.7 (9 – 48)	≥ 43	250 (28.3%)	635 (72.9%)
2: Subjective Norm	5	5–30	848	17.9 ± 5.7 (5 – 30)	≥ 22	237 (27.9%)	611 (72.1%)
3: Perceived behavioural control	6	6–36	887	27.5 ± 5.8 (6 – 36)	≥ 32	234 (26.4%)	653 (73.6%)
4: Decision making	3	3–18	877	12.7 ± 2.8 (3 – 18)	≥ 15	249 (28.4%)	628 (71.6%)
5: Intention and behaviour	4	4–24	882	15.1 ± 4.2 (4 – 24)	≥ 18	265 (30%)	617 (70%)

Abbreviations: *EBP* evidence based practice, *EBPI* Evidence-based Practice Inventory, *SD* Standard deviation

### Barriers and facilitators of EBP

The results of the regression analysis regarding facilitators and barriers for EBP adherence are detailed in Table 3. Determinants which demonstrated a positive influence towards EBP adherence in at least three dimensions, were: the availability of at least 60 min for scientific literature at work (OR 9.67; 95% CI: 5.86—16.30), followed by a master or higher academic degree (OR 9.09; 95% CI: 5.86—14.28), the drafting or involvement in at least one scientific publication (OR 7.06; 95% CI: 5.10—9.85), the hosting of EBP lectures (OR 7.02; 95% CI: 4.67—10.69), the availability of scientific literature at work (OR 4.49; 95% CI: 3.03—6.85), a weekly contact time of up to 10 h (OR 2.49; 95% CI: 1.58—3.93) and a work experience of 26–35 years (OR 2.58; 95% CI: 1.55—4.29).

**Table 3:** Association between EBP and socio-demographic factors analysed via exploratory univariate logistic regression analyses

	**Dimension 1** **Attitude**	**Dimension 2** **Subjective norm**	**Dimension 3** **Perceived behavioural control**	**Dimension 4** **Decision making**	**Dimension 5** **Intention and behaviour**
	Odds ratio [95% CI]				
**Highest professional degree**^a^ (*n* = 877)^c^					
Undergraduate (*n* = 90)	0.69 [0.37–1.24]	1.07 [0.61–1.80]	1.17 [0.59–2.17]	1.32 [0.77–2.23]	1.03 [0.57–1.78]
Diploma (vocational school) (*n* = 368)	Reference	Reference	Reference	Reference	Reference
Bachelor/diploma (university) (*n* = 273)	1.44 [1.01–2.06]	0.83 [0.56–1.21]	2.82 [1.91–4.20]	1.46 [1.02–2.09]	1.87 [1.31–2.68]
Master or higher academic degree (*n* = 146)	3.11 [2.08–4.68]	2.65 [1.77–3.99]	9.09 [5.86–14.28]	2.75 [1.83–4.15]	4.35 [2.89–6.59]
**Contact time with patients**^a^ (*n* = 861)^c^					
0 – 10 h/week (*n* = 120)	2.49 [1.58–3.93]	1.67 [1.04–2.66]	2.24 [1.41–3.56]	1.46 [0.90–2.33]	2.02 [1.30–3.16]
11 – 20 h/week (*n* = 180)	1.37 [0.90–2.09]	0.90 [0.57–1.41]	1.42 [0.92–2.19]	1.24 [0.81–1.90]	0.70 [0.45–1.07]
21 – 30 h/week (*n* = 242)	1.03 [0.69–1.54]	0.97 [0.65–1.46]	1.05 [0.69–1.58]	1.22 [0.82–1.81]	0.80 [0.54–1.17]
31- 40 h/week (*n* = 274)	Reference	Reference	Reference	Reference	Reference
> 40 h/week (*n* = 45)	0.90 [0.40–1.86]	2.37 [1.20–4.62]	0.64 [0.25–1.43]	1.09 [0.51–2.17]	0.83 [0.39–1.64]
**Primary setting of work**^b^ (*n* = 783)^c^					
Inpatient/hospital (*n* = 315)	Reference	Reference	Reference	Reference	Reference
Outpatient (*n* = 468)	0.97 [0.71–1.34]	0.95 [0.69–1.32]	0.97 [0.70–1.35]	0.96 [0.69–1.32]	1.11 [0.81–1.53]
**Work experience**^**a**^ (*n* = 870)^c^					
0 – 5 years (*n* = 278)	Reference	Reference	Reference	Reference	Reference
6 – 15 years (*n* = 273)	1.19 [0.82–1.72]	2.57 [1.73–3.87]	1.46 [0.99–2.15]	1.06 [0.72–1.55]	1.74 [1.20–2.54]
16 – 25 years (*n* = 172)	1.02 [0.66–1.55]	1.91 [1.20–3.04]	1.22 [0.78–1.90]	1.25 [0.81–1.92]	1.49 [0.97–2.29]
26 – 35 years (*n* = 120)	0.93 [0.56–1.50]	2.58 [1.55–4.29]	1.73 [1.07–2.79]	1.72 [1.08–2.73]	1.66 [1.03–2.67]
> 35 years (*n* = 27)	0.75 [0.27–1.83]	2.40 [0.93–5.81]	1.25 [0.47–2.96]	1.57 [0.64–3.60]	1.66 [0.68–3.80]
**Size of the city/municipality of employment**^b^ (*n* = 879)^c^					
≥ 20,000 inhabitants (*n* = 662)	Reference	Reference	Reference	Reference	Reference
< 20,000 inhabitants (*n* = 217)	0.57 [0.39–0.83]	0.81 [0.56–1.15]	0.64 [0.44–0.92]	0.61 [0.42–0.87]	0.76 [0.53–1.07]
**Hosting of lectures or workshops on EBP**^b^ (*n* = 885)^c^					
No (*n* = 765)	Reference	Reference	Reference	Reference	Reference
Yes (*n* = 120)	3.68 [2.48–5.48]	3.18 [2.54–5.73]	7.02 [4.67–10.69]	3.63 [2.44–5.41]	6.57 [4.36–10.06]
**Availability of scientific literature at work place**^b^ (*n* = 869)^c^					
No (*n* = 291)	Reference	Reference	Reference	Reference	Reference
Yes (*n* = 578)	1.79 [1.3–2.51]	4.49 [3.03–6.85]	1.63 [1.17–2.29]	1.31 [0.96–1.81]	1.28 [0.94–1.76]
**Drafting of or involvement in ≥ 1 scientific publication**^b^ (*n* = 887)^c^					
No (*n* = 610)	Reference	Reference	Reference	Reference	Reference
Yes (*n* = 277)	2.94 [2.16–4.00]	2.56 [1.87–3.50]	7.06 [5.10–9.85]	2.55 [1.87–3.46]	4.08 [3.01–5.57]
**Available time for scientific literature studies at work**^a^ (*n* = 751)^c^					
0 min/week (*n* = 272)	Reference	Reference	Reference	Reference	Reference
1 – 30 min/week (*n* = 207)	0.78 [0.51–1.18]	2.55 [1.57–4.20]	0.66 [0.41–1.04]	0.82 [0.54–1.26]	0.72 [0.47–1.10]
31 – 60 min/week (*n* = 137)	0.98 [0.62–1.55]	3.69 [2.20–6.27]	1.04 [0.64–1.68]	1.04 [0.65–1.65]	1.02 [0.64–1.61]
> 60 min/week (*n* = 135)	1.66 [1.07–2.56]	9.67 [5.86–16.30]	3.20 [2.07–4.98]	2.55 [1.65–3.94]	3.25 [2.12–5.02]

^a^OR calculated using a variable of the characteristic as reference

^b^OR calculated as the ratio between the odds in the presence of characteristic variable against the odds in the absence of the variable

^c^The sample size can differ within single dimensions because of missing values in the dependent variable

The primary setting of work did not show to be a determinant regarding EBP adherence across all dimensions, whereas a smaller size of the city/municipality of employment demonstrated to have a negative influence towards EBP adherence in dimensions 1, 3 and 4 (see Table 3 for details).

## Discussion

The consideration of EBP principles in clinical practice depends on the healthcare professional’s willingness and ability to combine the current best research evidence with patient preferences and his/her clinical experience.

This study aimed to describe the EBP adherence of healthcare professionals working in Germany and to explore barriers and facilitators to the implementation of and adherence to EBP. This is, to our knowledge, the first study to provide comprehensive evidence concerning the EBP adherence across healthcare professionals in Germany.

### EBP adherence

Our results showed that EBP adherence varied considerably across dimensions. In dimension 1 (attitude) and 3 (perceived behavioural control) more than 50% of the participants demonstrated a marked adherence to EBP, whereas in other dimensions, participants were more ambivalent (e.g., dimension 2 – subjective norm).

The findings from this study are in line with results from other international research findings. In 2011, Heiwe et al. [27] assessed EBP adherence and knowledge among Swedish healthcare professionals. Their findings showed positive attitudes towards EBP and its use to support clinical decision-making. Although literature and research findings were rated as useful in clinical practice, EBP was not perceived as taking into account the patient and healthcare professionals’ preferences. This finding mirrors a major criticism of EBP in general: the insufficient appreciation of the patients’ preferences is criticised as probably the most difficult and poorly mapped facet of EBP and was considered to receive the least attention in EBP research [28], which was explicitly not intended by Sackett et al. [29]. Hoffmann et al. [30, 31] advocated the incorporation of a shared decision making skills training into the actual EBP training for medical and health care students as a potential means to overcome this discrepancy in clinical practice.

### Facilitators and barriers

According to the literature, a major influencing factor regarding the adherence to and implementation of EBP is the healthcare professional’s individual attitude towards EBP [32]. With reference to the answers to dimension 1, the overall attitude of the current study sample towards EBP, was positive. However, still 20 to 30% were of the opinion that EBP does not sufficiently account for their clinical experience and individual differences between patients. This indicates an incomplete understanding of the EBP construct, which clearly incorporates external evidence with patient’s preferences and clinician’s individual expertise [1]. An academic training [33] or EBP workshops [34] could help to improve a healthcare professional’s knowledge of EBP principles and hence reduce current misconceptions.

To ensure sound clinical decision making, research evidence must be transformed into healthcare professional’s knowledge [1]. Regarding answers to dimension 3, participants in this study generally assessed themselves as being able to enact EBP in clinical practice, although keeping up with latest research evidence from the literature was stated to be the most challenging aspect (question 20). As most respondents reported to feel able to apply the 5 steps of EBP (questions 14 to 19), this inability needs to be further investigated, as external factors, such as lack of time and/or internet access could explain this issue and were reported in the literature to be barriers to EBP in clinical practice [35]. However, 52% (*n* = 458) of the respondents from the current study sample had a diploma or bachelor degree as highest professional degree. Therefore, the general self-reporting of being able to enact all 5 steps of EBP in clinical practice should be viewed cautiously, as it could also be estimated that some clinicians might not be interested in acquiring the necessary level of training to critically appraise research evidence [36]. However, Dawes et al. [1] stated that not all health care professionals needed to be able to appraise evidence from the ground up. Instead, individuals on different levels of responsibility within an organisation needed varying levels of sophistication in appraising research evidence. Managers and educators should use their critical appraisal skills to aim at producing more comprehensive and easy-to-access pre-appraised EBP material to inform ‘EBP users’, which could be seen as an additional strategy to ensure EBP in clinical practice [1, 36].

Although 41% of the respondents in our study sample reported to have a diploma from vocational school to be their highest professional degree and hence did not necessarily receive a curricularly established EBP training, 28% favourably evaluated EBP (sum scores across dimension 1). This is in keeping with findings from previous international studies of a variety of healthcare professionals [22, 26, 37, 38], which support the general positive attitude of healthcare professionals towards EBP. Yahui et al. [22] and Jette et al. [26] reported that physical therapists in Malaysia and the USA, respectively, had a positive attitude towards EBP and are inclined towards implementing evidence into their clinical practice. Philibert et al. [37] also reported that American occupational therapists generally had a positive attitude regarding EBP although ratings regarding the usefulness of research to inform clinical practice were less favourable. In the study by Knops et al. [38], Dutch surgeons and nurses also had a generally positive attitude towards EBP and were familiar with the EBP terminology, although more frequent staff meetings for the critical appraisal of research evidence was advocated.

### Regression analysis—determinants

The most important influencing factor of EBP adherence found in this study was the time available for studying or reading scientific literature at work. According to our results, at least one hour for studying scientific literature at work significantly increases a healthcare professionals’ chance to improve his/her own estimate of the social pressure to perform an EBP conform behaviour. These findings are in line with results of previous studies of American occupational and physical therapists [26, 37]. In the study by Jette et al. [26], 46% of the respondents named insufficient time as the most important barrier to the use of EBP in practice. Philibert et al. [37] reported time constraints to be the most important barrier for American occupational therapists to read scholarly journals. In 2006, [39], the study results by Upton and Upton showed that healthcare professionals across 14 different professional groups, such as speech and language therapists and psychologists, reported lack of both, time and money as main barrier to the implementation of EBP. Hence, considerable attention needs to be paid to further develop a German healthcare infrastructure, which is committed to best practice [1], requiring health policy action such as the implementation of a financial compensation system to offset the time invested for scientific literature at work [40].

Based on our results, persons with a higher academic degree were more likely to adhere to EBP. Dawes et al. [41] claimed that professions and their educational institutions need to incorporate the necessary knowledge, skills, and attitudes of EBP into their training and registration requirements. However, in Germany, currently only midwifery is legally established as a primary qualifying university degree course [10] among allied healthcare professions, constituting the principal legal stipulations to provide students with a basic understanding and level of EBP capability upon graduation [42]. As, for example, the German Training and Examination Regulations for Physical therapists (PhysTh-AprV) currently do not stipulate EBP training components, German physiotherapy education was stated not to meet the World Congress of Physiotherapy’s expectations regarding educational standards, which was considered a crucial issue to be addressed in the current debate on health policy [43]. However, our study sample also comprised professions trained in higher education institutions, such as medicine, psychology, and sports sciences, but they only represented 7% of the total sample (*n* = 63). The highest chances across all dimensions to adhere to EBP were shown to be associated with a postgraduate or higher academic degree, mirroring the fact that a mere university degree does not automatically implicate EBP adherence in clinical practice. These findings are in line with international requirements for medical training curricula which should enable their graduates’to have the ability to adapt to changing circumstances throughout their professional life’ [44]. An early claim by Shin et al. [45] supports the notion that in order to 'futureproof' healthcare graduates, they need to be trained in the necessary skills to support life-long learning through all the steps of EBP.

However, for those healthcare professionals who need to improve their EBP skills in clinical practice, the way with which to achieve this goal remains unclear. The design of effective training programmes, fostering not only healthcare professional’s attitudes and knowledge about EBP but also its implementation into clinical practice, is still matter to ongoing research [46–48]. In a systematic review from 2016, Hecht et al. [49] reported insufficient evidence as to whether currently available EBP trainings for healthcare professionals resulted in a meaningful change in EBP behaviour in clinical practice. However, in a recent study by Draaisma et al. [50] the authors showed that healthcare professionals who were exposed to deliberate practice of EBP in a routine clinical setting, evaluated EBP as more useful and were more likely to use it in decision making than their peers who only followed a standard EBP workshop.

Participants who stated to have drafted or have been involved in at least one scientific publication or have hosted lectures and workshops on EBP, felt more able to enable EBP behaviour (dimension 3). Hosting lectures as well as drafting a scientific publication presupposes a thorough understanding of the complex construct of EBP as well as comprehensive skills regarding its teaching [51]. However, both aspects may not directly mirror a person’s ability to adhere to EBP in clinical practice. Kaper et al. [4] noted that the EBPI does not concern step 4 (application) and 5 (audit) of the EBP process but explicitly assesses attitude, behaviour, information processing, decision making, and department setting conditions via self-report. Hence, both these factors need to be further assessed as to whether they actually influence a person’s adherence to EBP in clinical practice by means of qualitative [52] or mixed methods research approaches [53, 54], going beyond the sole self-report of EBP adherence.

### Study strengths and limitations

A major limitation of this study, as already published elsewhere [14, 55], might have been the issue of sampling bias, mirrored by the above mentioned relatively low response rate of 13% (according to the number of accesses to the survey homepage). We strived to include a representative sample in the online survey by employing different media to bring the survey to the community of German healthcare professional’s attention. Yet, with respect to the total number of healthcare professionals working in Germany, the total number of participants (*n* = 889) was relatively low [14]. Currently, there are approximately 385,100 active physicians (in 2017) [56], approximately 1,064,342 healthcare professionals who are subjected to social insurance contributions (in 2018) [57] and approximately 192,000 physical therapists (in 2016) [58] working in Germany [14]. Therefore, we must presume that most healthcare professionals in Germany were not aware of the survey or decided not to participate, although we tried to distribute the information about the survey as broadly as possible [14]. In particular, medical physicians and nurses were not sufficiently represented [14]. A wider announcement of the survey among these professionals as well as the utilization of the Total Design Method provided by D.A. Dillman [59] as well as incentives might have increased the rate of participation [14, 55].

A further limitation was the sole online accessibility of the survey [14, 55]. This fact might have inflated the participation of (younger) healthcare professionals and people familiar with the use of digital media and online contents. In contrast, it might have prevented (older) people from participation, who are not that familiar with online content or healthcare professionals working in institutions without internet access [14]. However, the mean age of participants (37.4 years) in our study was lower than that of the total working population in Germany (43 years) [14, 55, 60]. However, representative data for the age distributions of healthcare professionals working in Germany are not available. In addition, younger healthcare professionals may be distinct from older (more experienced) ones regarding their EPB adherence, as stated by Dysart et al. [61], who reported greater reservation towards research evidence among more experienced occupational therapists compared to less experienced colleagues [14, 55].

In addition, our study was conducted as a self-administered online survey, not as an actual audit of current clinical practice. This might not provide a complete realistic impression of EBP under routine clinical care conditions [62].

Currently, there is no standardised instrument to assess the construct of EBP in its complexity, hampering the comparability of results across studies. In 2014, a systematic review by Fernándes-Dominguez et al. [63] identified 24 instruments assessing EBP adherence as well as barriers and facilitators only among the professional group of physical therapists with all instruments judged to lack sufficient comprehensiveness regarding the validation procedure. We used a cross-culturally adapted German version of the EBPI, an instrument with evidence of sufficient reliability [14] and construct validity [4].

Despite these limitations, our study presents several strengths. This is the first survey to systematically assess self-reported adherence to EBP across healthcare professionals in Germany. In addition, we also explored a broad set of potential facilitators and barriers regarding EBP implementation into clinical practice, which constitutes a crucial component for implementing research evidence into routine clinical practice [62].

Although our sample size did not arrive to be representative for the entire group of healthcare professionals in Germany, we reached 889 respondents across 9 professions, which is comparable to other international nationwide surveys [39] across healthcare professionals [14].

Regarding the univariate logistic regression analysis, we decided to draw influencing factors which were previously used [4] in similar studies, which constitutes a sound methodological approach recommended for exploratory regression analysis [15].

A further strength of this study was the use of the “snowball-principle”, which led to the involvement of many national journals, newspapers, professional societies, informal social media groups and other ways to distribute the survey nationwide to as many potential participants as possible [14]. All healthcare professionals working in Germany were able to access our survey online without any preceding restrictions, such as a password [14, 55].

## Conclusion

The significance of our study is based on the focus on assessing EBP adherence across different healthcare professions in Germany. We used a validated and established questionnaire to assess various dimensions of EBP adherence in various clinical settings. Our results demonstrated that most respondents had a generally positive attitude towards EBP and considered it an important aspect of high-quality care. Respondents were more cautious in how far EBP respects individual differences between patients or leaves them autonomous in their individual clinical decision, mirroring an incomplete understanding of EBP. However, EBP and clinical guidelines were both rated as helpful for making better clinical decisions. Our data showed that, although training pathways are distinct from international standards, German healthcare professionals experience similar barriers towards the implementation of EBP in clinical practice. Major influencing factors for the adherence to EBP identified were the time for scientific literature at work, the (highest) professional degree, as well as the involvement in drafting scientific publications or hosting EBP workshops. Our results were in agreement with findings from previous international studies investigating EBP adherence among healthcare professionals with various academic educational biographies. Some of the barriers to EBP adherence were identified to be work place related, such as the time for and availability of scientific literature at work, requiring changes to the current German healthcare infrastructure. Other factors, such as a higher academic degree, are more related to the underlying German healthcare educational system, which modification would warrant a change in the German educational policy of healthcare professionals.

## Supplementary Information

**Additional file 1.** Online Survey (in English language).

**Additional file 2.** Media used to inform the community of healthcare professionals in Germany about the online survey.

## Data Availability

The datasets used and/or analysed is this study are available from the corresponding author upon reasonable request.

## Abbreviations

CHERRIES: Checklist for Reporting Results of Internet E-Surveys
CI: Confidence interval
EBP: Evidence-based practice
EBPI: Evidence-based Practice Inventory
OR: Odds ratio
PhysTh-AprV: Ausbildungs- und Prüfungsverordnung für Physiotherapeuten (German Training and Examination Regulations for Physical therapists)
SAMPL: Guideline for Statistical Analyses and Methods in the Published Literature’
STROBE: Checklist for Strengthening the Reporting of Observational Studies in Epidemiology
