# Supplementary material for: Adherence to evidence-based practice across healthcare professionals in Germany: results from a cross-sectional, nationwide survey
Source: BMC Health Serv Res. 2022 Oct 25;22:1285. doi: 10.1186/s12913-022-08682-z (PMC9597997; doi:10.1186/s12913-022-08682-z)
Supplement: Supplementary file 1 — Additional file 1. Online Survey (in English language). [file 12913_2022_8682_MOESM1_ESM.pdf]

0% ausgefüllt

Germany-wide survey on facilitating factors and barriers in relation to evidence-based practice

Dear Sir or Madam,
How do people in the health care system in Germany make decisions and what influences these decisions?

In cooperation with the German Society for Physical Therapy Science (DGPTW) e. V., the University of Applied Health Sciences Bochum is conducting a survey to investigate barriers and facilitating factors in the implementation of evidence-based practice. The term evidence-based practice describes a problem-solving approach to making clinical decisions, incorporating current best research evidence, clinical experience and the characteristics, preferences and values of the individual patient.
We are conducting a nationwide, anonymous, voluntary survey on this topic. Members of various health care professions (e.g., occupational therapy, midwifery, medicine, nursing, physical therapy, psychology, sports therapy, speech therapy) will be surveyed.
The results can help understand how and why patient care decisions are made. At the same time, based on this survey, it may be possible to infer an existing need in terms of strengthening enabling factors as well as reducing existing barriers.
Our goal with this survey is to help improve patient care and we hope you have approximately 10 minutes to participate in this survey.
Please participate in the survey only once, and please forward the survey link to your colleagues as well.
With collegial regards,

Prof. Dr. Christian Kopkow, MPH	Prof. Dr. Kerstin Lüdtke
Studienleitung	1. Vorsitzende der DGPTW e. V.

Legend:
KL and CK as board members of the German Society of Physical Therapy Research (DGPTW) e.V. granted permission to use the DGPTW Logo for the purpose of this research project.

Weiter

7% ausgefüllt

Participant information

What is the aim of the study?

The purpose of the study is to assess facilitators and barriers to evidence-based practice among members of various health care professions (e.g., occupational therapy, midwifery, medicine, nursing, physical therapy, psychology, sports therapy, speech therapy). Another aim of this study is to test whether the questionnaire used is reliable, i.e. whether the questionnaire gives the same result at different time points.

What will be investigated?
In order to achieve the study aims, we would like to ask you some questions about the following topics:
Facilitating factors and barriers in the use of evidence-based practice.
Demographics

How time consuming is the participation?
Participation in the study (online survey) will take approximately 10 minutes.
If you agree, we will invite you a second time 2 weeks after initial participation to take the survey (to check the reliability of the questionnaire). However, this is only if you want to - you are also welcome to participate in the survey only once.

What happens with my data?
The collected data will be kept under lock and key and will only be published in anonymized form for scientific purposes. Anonymization is the alteration of personal data in such a way that the individual details about personal or factual circumstances can no longer be attributed to a specific or identifiable natural person, or can only be attributed to a specific or identifiable natural person with a disproportionate amount of time, cost and effort (Section 3 (6) BDSG). In the case of publication of study results, personal identification is not possible. Primary data as the basis for publications should be stored on durable and secure carriers at the institution where they originated for ten years. After that, these data will be deleted, unless there are legal and statutory retention periods to the contrary.

What are the risks?
There are no risks associated with the survey. Your participation in this study is voluntary. You can withdraw your participation at any time without giving reasons and without incurring any disadvantages. You are not required to answer every question and may stop the survey at any time.
Please contact the principal investigator, Prof. Dr. Christian Kopkow, if you have any further questions about the study or if you have not understood anything in this participant information.
Prof. Dr. Christian Kopkow can be reached at:
Phone: 0234 - 777 27 624
E-mail: christian.kopkow@hs-gesundheit.de
Thank you for your participation,
the study team of the Hochschule für Gesundheit

Zurück Weiter

14% ausgefüllt

Privacy policy

- I agree that the data collected in the course of the study may be recorded on electronic data carriers and stored in anonymized form at the survey and evaluation center for scientific evaluation.
- Furthermore, I agree that an authorized representative of the Ethics Committee, who is bound to secrecy, may inspect my personal data available at the Survey and Evaluation Center, as far as this is necessary for the review of the study. For this measure, I release the survey and evaluation center from the duty of confidentiality.
- I know that I can revoke my consent at any time without incurring any disadvantages.
- Anonymization is the alteration of personal data in such a way that the information about personal or factual circumstances can no longer be assigned to a specific or identifiable natural person, or can only be assigned to such a person with a disproportionately large effort in terms of time, costs and manpower(§ 3 para.6 BOSG).

I have read and understood the participant information for the study 'Intercultural adaptation and psychometric evaluation of the German version of the Evidence-based practice inventory'. I hereby give my consent to participate in the aforementioned study and agree to the recording of my data and its disclosure as well as to the inspection of my personal data in the form described above.

You must click "**Continue**" to participate in the survey.

Prof.Dr.Christian Kopkow,
Hochschule für Gesundheit Bochum,
Department of Applied Health Sciences,
Gesundheitscampus 6-8, 44801Bochum

Zurück

Weiter

21% ausgefüllt

Important note for answering the questions
As a clinician, you make many clinical decisions for your patients every day. This questionnaire is designed to illustrate the process of how clinicians arrive at their clinical decisions in daily practice. The answers to the questions below should reflect the way you typically make decisions. We are asking you to provide your personal view within the context of this questionnaire. There are no right or wrong answers.
Thank you for participating in the survey. Your responses are anonymous and will be kept confidential.
However, before proceeding to answer the questions, please read the following definitions carefully. These definitions are important for understanding and correctly answering the following questions.

Note: For ease of reading, the masculine form of speech is used throughout this questionnaire for personal nouns and pronouns. However, this does not imply any discrimination against the female gender, but is intended to be understood as gender-neutral for the sake of linguistic simplification.

Clinician	The term "clinician" in this questionnaire includes all health care professionals, such as physicians, therapists, nurses, psychologists, or midwives.
Patient	The term "patient" includes all persons who use a health-related service. This also includes healthy persons (e.g. expectant mothers, persons in primary prevention) or persons designated as clients or customers by certain professional groups.
Evidence	In the context of evidence-based medicine, the term "evidence" is derived from the English word "evidence " (= statement, , proof, result) and refers to the information from scientific studies and systematically compiled clinical experience that corroborates or refutes a fact.
Evidence Based Practice (EBP)	Problem-solving approach to making clinical decisions, incorporating current best research evidence, clinical experience, and the characteristics, preferences, and values of the individual patient(s).
Clinical decision	Deciding what actions to take in patient care after weighing information about alternative options.
Guideline	Guidelines are systematically developed, scientifically based and practice-oriented decision-making aids. They are intended to assist healthcare professionals in making decisions regarding diagnosis, therapy, or related clinical issues.
Quantitative Information	"Quantitative" means "relating to quantity", i.e. the number, size or quantity of something. Quantitative information is information that has a quantitative and/or numerical character, e.g. is represented as number/size/quantity.

Note: Move the mouse pointer over the selected words in the course of the questionnaire to display the definition again.

Zurück | Weiter

29% ausgefüllt

I. Demographic information:

1. How old are you? _____years ☐ not specified

2. What gender are you? ☐ male ☐ female ☐ other ☐ not specified

3. What health care profession do you belong to? (Multiple answers possible)

☐ occupational therapy ☐ midwifery ☐ speech therapy ☐ medicine ☐ nursing ☐ physiotherapy ☐psychology

☐ ☐ Sports Science ☐ other

☐ not specified

4. Which of the following degrees have you already earned in the health field? (Multiple answers possible)

☐ currenty in training ☐ Diploma ☐ University Degree ☐ Bachelor degree

☐ Master's degree ☐ PhD ☐ other ☐ not specified

Zurück Weiter

29% ausgefüllt

5. When you think about a typical work week, how much time do you spend in direct patient contact?
Approximately _______ hours ☐ not specified

6. What is your current work environment? (Multiple answers possible)
☐ hospital ☐ university hospital ☐ Rehabilitation clinic ☐ practice ☐ other____________ ☐ not specified

7. In which state do you work?
☐ Lower Saxony ☐ North-Rhine Westphalia ☐ Rhineland-Palatinate
☐ Saarland ☐ Thuringia ☐ Hessia ☐Baden-Wuerttenberg ☐ Bavaria ☐ Saxony-Anhalt ☐ Saxony
☐ Brandenburg ☐ Mecklenburg-Western Pomerania ☐ Schleswig-Holstein ☐ Hamburg ☐ Berlin ☐ Bremen
☐ not specified

8. How many years of clinical work experience do you have?
_____________years (rounded to full years after "vocational training") ☐ not specified

9. Are you?
(If you have more than one employment relationship, please answer this question in relation to the employment relationship with the highest volume).

☐ Employee
☐ Self-employed
☐ Free-lance
☐ in training/study/ practical year or similar

☐ not specified

Zurück Weiter

10. Are you employed in a managerial position? (defined as a person with personnel responsibility)

☐ yes ☐ no ☐ not specified

11. Are you in exchange with other health care professions (physicians, psychologists, occupational therapists, ...) in your facility or professional environment/field of activity?

☐ no ☐ yes ☐ not specified

Zurück Weiter

12. How many inhabitants live in the city (or municipality) where your workplace is located?
(If you have more than one job, please answer this question in relation to the job where you work the most hours).
☐ <5,000 inhabitants (rural community)
☐ 5,000 - 20,000 inhabitants (small town)
☐ 20,000 - 100,000 inhabitants (medium-sized city)
☐ >100,000 inhabitants (large city)
☐ not specified

13. In a typical work week, how much time do you have available at work to engage with information from scientific publications?
______minutes
☐ not specified

14. Do you have the possibility to access evidence-based practice publications at your workplace?
☐ yes ☐ no ☐ not specified

15. Have you already written or collaborated on one or more scientific publications?
☐ yes ☐ no ☐ not specified

16. Do you lecture on, or offer workshops in evidence-based practice?
☐ yes ☐ no ☐ not specified

Zurück Weiter

II. Evidence-based practice inventory

Now please read each statement carefully and select a numerical value for each statement that most closely reflects your experience/attitude.

Note: As you go through the questionnaire, hover your mouse over the highlighted words to redisplay the definition.

1. I feel that EBP is **useless**	①②③④⑤⑥	**useful** to improve my patients' outcomes.
2. I feel that EBP is an **unimportant**	①②③④⑤⑥	**important** feature of high-quality patient care.
3. I feel that EBP **worsens**	①②③④⑤⑥	**improves** the quality of my clinical decisions.
4. I feel that EBP **disregards**	①②③④⑤⑥	**respects** my clinical experience.
5. I feel that EBP **disregards**	①②③④⑤⑥	**respects** individual differences between my patients.
6. EBP makes me feel **constrained**	①②③④⑤⑥	**autonomous** in my clinical decisions.
7. EBP **hinders**	①②③④⑤⑥	**helps** me in making better clinical decisions.
8. I feel that clinical guidelines in my own discipline **hinder**	①②③④⑤⑥	**help** me in making decisions.

Zurück Weiter

Note: As you go through the questionnaire, hover your mouse over the highlighted words to redisplay the definition.

9. My colleagues **discourage**	①②③④⑤⑥	**encourage** me to apply EBP principles in my clinical decisions.
10. In my department, we **pay no**	①②③④⑤⑥	**a lot of attention** to applying EBP principles in our clinical decisions.
11. Managers in my department **hinder**	①②③④⑤⑥	**support** me to apply EBP principles in my clinical decisions.
12. My colleagues and I **rarely**	①②③④⑤⑥	**frequently** discuss and challenge how we make our clinical decisions.
13. My colleagues and I **rarely**	①②③④⑤⑥	**frequently** discuss research evidence from literature.

Zurück Weiter

Note: As you go through the questionnaire, hover your mouse over the highlighted words to redisplay the definition.

14. I feel that I am **incapable**	①②③④⑤⑥	**capable** of applying EBP principles in my clinical decisions.
15. I feel that I am **incapable**	①②③④⑤⑥	**capable** of translating my information needs into relevant and feasible clinical questions.
16. I feel that I am **incapable**	①②③④⑤⑥	**capable** of searching for research evidence in literature.
17. I feel that I am **incapable**	①②③④⑤⑥	**capable** of critically appraising research evidence from literature.
18. I feel that I am **incapable**	①②③④⑤⑥	**capable** of translating research evidence to the care of my individual patients.
19. I feel **incapable**	①②③④⑤⑥	**capable** of regularly keeping up with latest research evidence from literature.

Zurück Weiter

71% ausgefüllt

Note: As you go through the questionnaire, hover your mouse over the highlighted words to redisplay the definition.

20. I give **low**	①②③④⑤⑥	**high** priority to a thorough understanding of the background of the answers to my clinical questions.
21. I **dislike**	①②③④⑤⑥	**like** using numbers, tables, and other quantitative information for supporting my clinical decisions.
22. When making clinical decisions, I prefer to use my **intuition and experience**	①②③④⑤⑥	**facts and arguments**.

Zurück

Weiter

79% ausgefüllt

Note: As you go through the questionnaire, hover your mouse over the highlighted words to redisplay the definition.

23. I **rarely**	①②③④⑤⑥	**frequently** use research evidence to support my clinical decisions.
24. I prefer to use **my own experience**	①②③④⑤⑥	**research evidence** for making my clinical decisions.
25. I tend to **ask colleagues**	①②③④⑤⑥	**search the literature** to find answers to my clinical questions.
26. I **rarely** clinical	①②③④⑤⑥	**frequently** seek out available research evidence to answer my daily question.

Zurück Weiter

86% ausgefüllt

Finally, we ask you to provide your email address so that we can invite you to participate in the second survey by email in 2 weeks to check the reliability of the questionnaire. Your data will of course not be passed on to third parties and will be used exclusively for the purpose of this study.

E-Mail Adress:____

Zurück Weiter

Thank you very much for your participation!

We would like to thank you very much for your participation.
Your answers were stored. You can now close the browser window.

Zurück

Weiter
