# Supplementary material for: Adherence to evidence-based practice across healthcare professionals in Germany: results from a cross-sectional, nationwide survey
Source: BMC Health Serv Res. 2022 Oct 25;22:1285. doi: 10.1186/s12913-022-08682-z (PMC9597997; doi:10.1186/s12913-022-08682-z)
Supplement: Supplementary file 2 — Additional file 2. Media used to inform the community of healthcare professionals in Germany about the online survey. [file 12913_2022_8682_MOESM2_ESM.pdf]

Additional file 2: Media used to inform the community of healthcare professionals in Germany about the online survey

	Description	Homepage post	Facebook post	Twitter post	Press release	Newsletter[1]
**Universities/ Research Centers**	Hochschule für Gesundheit (University of Applied Sciences; host institution)	X	X	X	X	
	MedEcon Ruhr (German network for health economy in the Ruhr area)	X				
	Fakultät für Sportwissenschaften RUB (Faculty for sport sciences of the Ruhr University Bochum)		O			
**Societies**	Deutsches Netzwerk Evidenzbasierte Medizin (German network for evidence based medicine)	X				
	Deutscher Bundesverband für akademische Sprachtherapie und Logopädie e.V. (German federal association for academic speech therapy)	X				
	LOGO Deutschland e.V. (German association for self-employed speech therapists)	X				X
	Deutsche Gesellschaft für Physiotherapiewissenschaft e.V. (German society for physiotherapy science)	X	X	X		
	Physio-Deutschland e.V. (German association for physiotherapy)	X	X			
	Verband Physikalische Therapie e.V. (German association for physical therapy)	X				
	Deutscher Verband der Ergotherapeuten (German association of occupational therapists)		X			
	Bundesverband für Ergotherapeuten in Deutschland. e.V. (German federal association for occupational therapists)		X			
	Deutsche Gesellschaft für Hebammenwissenschaften e.V. (German society of midwifery science)	X				
	Hebammen NRW (German midwifery association North Rhine-Westphalia)		O			
	Hebammenlandesverband Bremen e.V. (German midwifery association bremen)	X	X			

	Deutscher Verband für Podologie (ZFD) e.V. (German association for podology)		O			
	Deutsche Gesellschaft für HNO-Heilkunde, Kopf- und Hals-Chirurgie e.V. (German society of oto-rhino-laryngology, head and neck surgery)	X				
	Gesellschaft zur wissenschaftlichen Untersuchung von Parawissenschaften (Society for the scientific investigation of parasciences)	X				
**Journals and Newspapers**	Medizin Aspekte (German online journal for healthcare professions)	X				
	idw - Informationsdienst Wissenschaft (Online service for scientific information and research)	X				
	Pflegewissenschaft (German journal for nursing sciences)	X				
	pt - Zeitschrift für Physiotherapeuten (German journal for physiotherapists)		O			
	Thieme liebt Physiotherapeuten (German Facebook group of a publisher for physiotherapy media and journals)		O			
	Focus Online Local (Local online news journal)	X				
	Lokalkompass (Local online news journal for the region of Bochum)	X				
	JuraForum (German online platform for legal issues)	X				
	Ärzte Zeitung (German journal for medical practitioners)		O			
**Informal groups in social media**	ErgoXchange (Facebook page of an online service for occupational therapist)		O			
	Physio meets science (Facebook page for evidence-based medicine in physiotherapy)		O			
	Sportwissenschaften.net (Facebook page for sport sciences and fitness)		O			
	Podologie und Fußpflege (Facebook group for podology and medical footcare)		C			
	Podologie und Podologen in Deutschland (Facebook group for podology in Germany)		C			
	Physiotherapie Deutschland (Facebook group for physiotherapists in Germany)		C			

X = the survey has been advertised/posted/communicated/reported/distributed in/via this media; O = post in an “open group” in a social media (the post is accessible to the public in this social media); C = post in a closed group in a social media (the post is visible for group members only)

[1] for subscribers
